# Nasopalatine Duct Cyst

**DOI:** 10.1155/2013/869516

**Published:** 2013-11-04

**Authors:** Pratik Dedhia, Shely Dedhia, Amol Dhokar, Ankit Desai

**Affiliations:** ^1^Department of Oral Medicine and Radiology, Terna Dental College, Nerul, Navi Mumbai 400706, India; ^2^Department of Pediatric and Preventive Dentistry, Nair Hospital Dental College, Mumbai Central, Mumbai 400008, India; ^3^Department of Periodontics, Terna Dental College, Nerul, Navi Mumbai 400706, India

## Abstract

The nasopalatine cyst is the most common epithelial and nonodontogenic cyst of the maxilla. The cyst originates from epithelial remnants from the nasopalatine duct. The cells may be activated spontaneously during life or are eventually stimulated by the irritating action of various agents (infection, etc.). It is different from a radicular cyst. The definite diagnosis should be based on clinical, radiological, and histopathological findings. The treatment is enucleation of the cystic tissue, and only in rare cases a marsupialisation needs to be performed. A case of a nasopalatine duct cyst in a 35-year-old male is reviewed. The typical radiologic and histological findings are presented.

## 1. Introduction

The nasopalatine duct cyst (NPDC) was first ever described by Meyer in 1914 [1, 2]. Nasopalatine duct cyst, also termed as incisive canal cyst, arises from embryogenic remnants of nasopalatine duct, the communication between the nasal cavity and anterior maxilla in the developing fetus. Most of these cysts develop in the midline of anterior maxilla near the incisive foramen [3]. It is one of the most common nonodontogenic cysts of the oral cavity occurring in about 1% of the population [4].

NPDCs affect a wide age range; however, most present in the fourth through sixth decades of life, and most studies show a significantly higher frequency in men than woman, with the ratio being 2.5 : 1 [5–11]. Patients may be asymptomatic, with the lesion being detected on routine radiographs; however, many will present with one or more symptoms. Complaints are often found to be associated with an infection of a previously asymptomatic nasopalatine duct cysts and consist primarily of swelling, drainage, and pain [10, 12]. The vitality of nearby teeth should not be affected; however, it is not uncommon to see evidence of endodontic therapy because the nasopalatine duct cyst was previously clinically misdiagnosed as a periapical cyst or granuloma.

The present case of NPDC is one such typical pathology with the classical presentation which could have been easily misdiagnosed as a periapical lesion.

## 2. Case Report

### 2.1. History

A 35-year-old male reported to the dental clinic with the chief complaint of painless swelling over the palate and anterior maxilla. The swelling was gradually increasing in size for the past 3 months with associated displacement of maxillary central incisors. There was no associated history of trauma.

**Figure 1:**
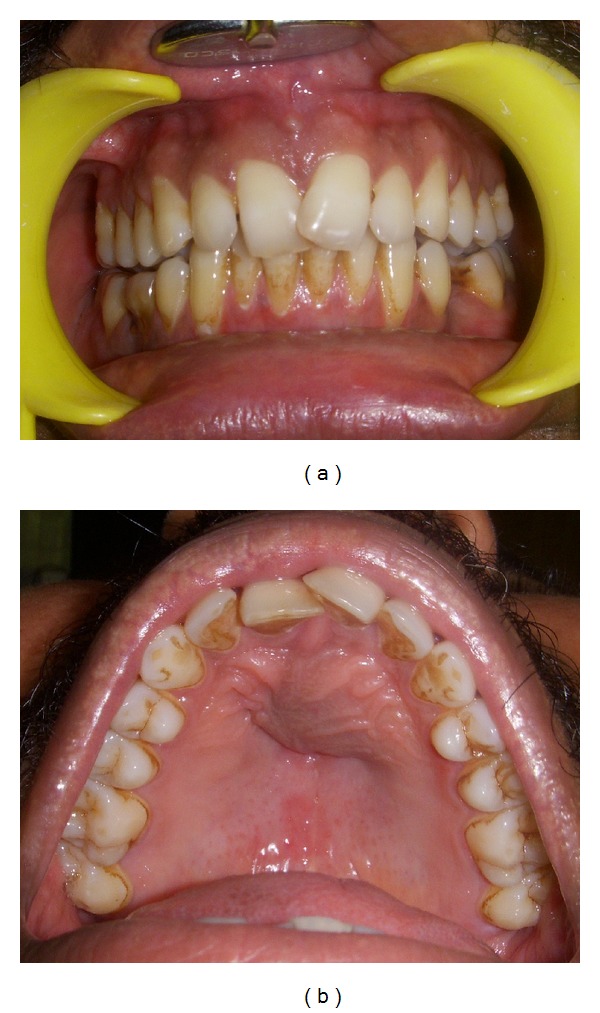
Preoperative intraoral swelling.

On examination, a well defined firm nontender swelling was seen on the left side of anterior hard palate and crossing over the midline to the right side. The crown of left central incisor was displaced labially and mesially, overlapping the crown of right central incisor (Figure 1). A provisional diagnosis of periapical cyst was given but the vitality tests of the central incisors were negative. The differential diagnosis was established with other conditions such as an nasopalatine duct cyst, enlarged nasopalatine duct, central giant cell granuloma, a root cyst associated to the upper central incisors, a supernumerary tooth follicular cyst, primordial cyst, and nasoalveolar cyst. Radiological investigations were subsequently advised.

### 2.2. Radiographic Features

**Figure 2:**
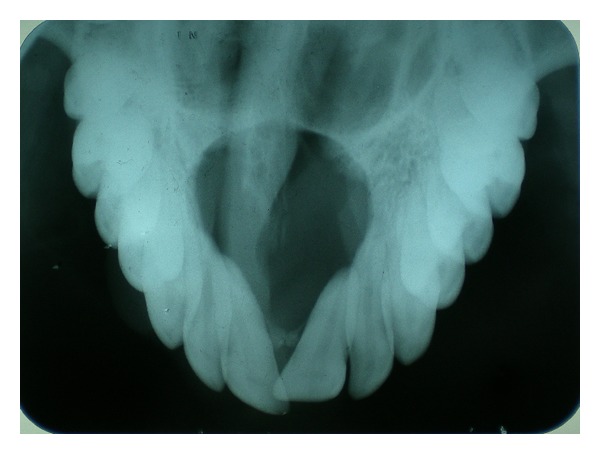
Maxillary occlusal view showing well defined radiolucency in the anterior maxilla.

Maxillary occlusal radiograph showed a well-defined round radiolucency approximately 2.5 cm in size with corticated margins in the midline and between the central incisors which was the typical radiographic feature of NPDC (Figure 2). The lesion was causing displacement of the roots of the incisors. Superiorly it was extending till the floor of nasal fossa. Also there was deviation of the nasal septum to the right side.

**Figure 3:**
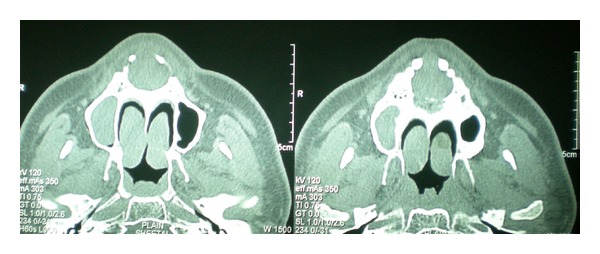
Axial sections of CT scan showing the well-defined round radiolucency.

**Figure 4:**
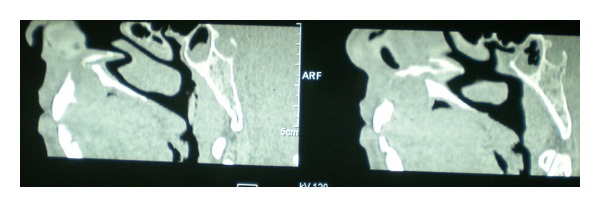
Sagittal sections of CT scan.

**Figure 5:**
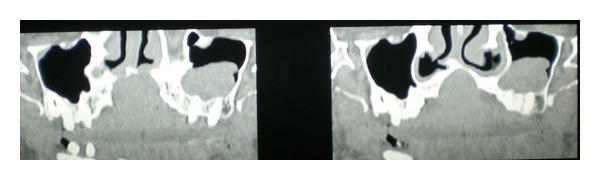
Panoramic reconstruction of CT scan.

CT scan also showed a well-defined radiolucency in anterior maxilla in the region of incisive canal, 2.6 cm × 2.8 cm in size. Mesiodistally the radiolucency was causing displacement of the roots of both maxillary central incisors and it was not extending laterally beyond the roots of incisors. Inferiorly it was extending till the crest of interdental bone. Loss of cortication was seen along the buccal and palatal aspects of the lesion in the sagittal sections. The lesion was causing mild elevation of the floor of nasal fossa in the anterior region with deviation of the nasal septum to the right side (Figures 3, 4, and 5). Mucosal polyp was seen associated with the floor of left maxillary sinus. A radiographic diagnosis of NPDC was established and surgical enucleation was planned.

### 2.3. Treatment

**Figure 6:**
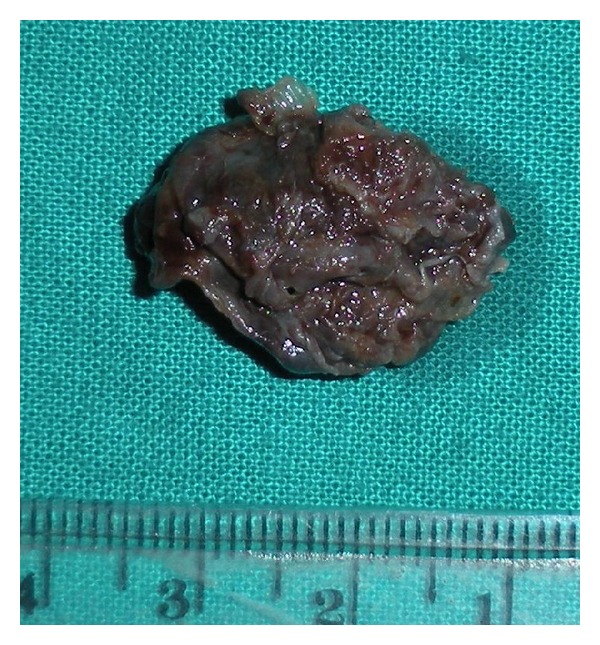
Gross specimen of the enucleated cyst.

**Figure 7:**
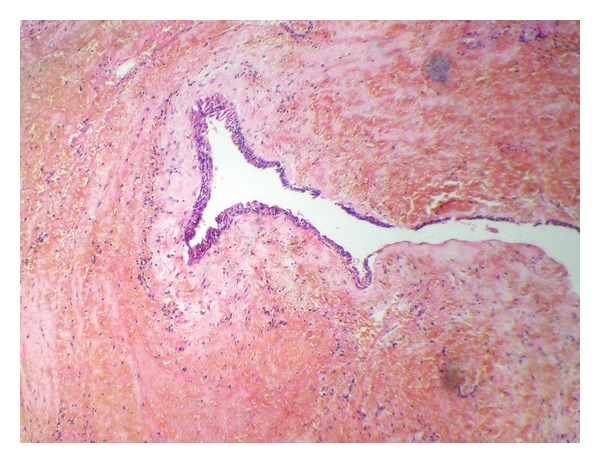
Photomicrograph of nasopalatine duct cyst showing stratified squamous epithelium.

A surgical enucleation was done with intact removal of the cyst (Figure 6) and the specimen was sent for histopathological examination which showed cystic lining composed of stratified squamous epithelium about 2-3 cell layer thick. Lining was flattened and showed pseudostratification at places (Figure 7). This was conclusive for NPDC.

## 3. Discussion

NPDCs are usually central or unilateral with no prevalence of side occurrence. Radiographically, they are seen as well-defined round or oval radiolucencies in the midline, although some lesions may appear heart-shaped [6], either because they become notched by the nasal septum during their expansion or because the nasal spine is superimposed on the radiolucent area.

Due to similar signs and symptoms, the NPDC may be easily misdiagnosed by the clinicians as a periapical lesion. This is why many authors believe that its prevalence is actually higher than presented in the literature [13]. Although a large NPDC might show the adjacent incisors roots to be within the cystic cavity, the lamina dura will be usually intact and the pulp usually vital, whereas a radicular cyst is associated with a pulpless tooth and involves a portion of the root, usually with loss of continuity of the lamina dura. In the reported case, the lesions were close to the apexes of the maxillary anterior teeth which were vital.

A reported 71.8% of NPDCs have squamous, columnar, cuboidal, or some combination of these epithelial types; respiratory epithelium is seen in only 9.8% [12, 14, 15]. In this case the squamous epithelium was present with stratification.

The present case had typical clinical, radiographic, and histopathological features of a nasopalatine duct cyst. The importance of this case is in the diagnosis of such lesions which can easily be misinterpreted as periapical cyst and inadvertent root canal therapy of surrounding vital teeth can be avoided.

## 4. Conclusion

Nasopalatine duct cysts are the most common nonodontogenic cyst of the oral cavity seen in the general population. NPDCs must be distinguished from other maxillary anterior radiolucencies. Vitality testing of teeth adjacent to or involved with a cyst-like lesion is mandatory and the final diagnosis could only be performed after histological analysis. It is important that practitioners are aware of the features of the NPDC.
